# The Effect of Different Boiling and Filtering Devices on the Concentration of Disinfection By-Products in Tap Water

**DOI:** 10.1155/2013/959480

**Published:** 2013-02-17

**Authors:** Glòria Carrasco-Turigas, Cristina M. Villanueva, Fernando Goñi, Panu Rantakokko, Mark J. Nieuwenhuijsen

**Affiliations:** ^1^Centre for Research in Environmental Epidemiology (CREAL), Barcelona Biomedical Research Park, Dr. Aiguader 88, 08003 Barcelona, Spain; ^2^CIBER Epidemiología y Salud Pública (CIBERESP), Instituto de Salud Carlos III, Melchor Fernández Almagro 3–5, 28029 Madrid, Spain; ^3^Gipuzkoa Laboratory of Public Health, Avenida de Navarra 4, 20013 San Sebastián, Spain; ^4^Neulanen Research Centre, National Institute for Health and Welfare (THL), Neulaniementie 4, 70701 Kuopio, Finland

## Abstract

Disinfection by-products (DBPs) are ubiquitous contaminants in tap drinking water with the potential to produce adverse health effects. Filtering and boiling tap water can lead to changes in the DBP concentrations and modify the exposure through ingestion. Changes in the concentration of 4 individual trihalomethanes (THM4) (chloroform (TCM), bromodichloromethane (BDCM), dibromochloromethane (DBCM), and bromoform (TBM)), MX, and bromate were tested when boiling and filtering high bromine-containing tap water from Barcelona. For filtering, we used a pitcher-type filter and a household reverse osmosis filter; for boiling, an electric kettle, a saucepan, and a microwave were used. Samples were taken before and after each treatment to determine the change in the DBP concentration. pH, conductivity, and free/total chlorine were also measured. A large decrease of THM4 (from 48% to 97%) and MX concentrations was observed for all experiments. Bromine-containing trihalomethanes were mostly eliminated when filtering while chloroform when boiling. There was a large decrease in the concentration of bromate with reverse osmosis, but there was a little effect in the other experiments. These findings suggest that the exposure to THM4 and MX through ingestion is reduced when using these household appliances, while the decrease of bromate is device dependent. This needs to be considered in the exposure assessment of the epidemiological studies.

## 1. Introduction 

Safe drinking water is a vital need for humans. The access to drinking water is becoming more constrained worldwide, by both growing demand and more erratic availability. Disinfection is necessary to have safe drinking water. However, undesired disinfection by-products (DBPs) are formed by reaction of one disinfectant, or a mixture of them, with organic matter. DBPs are ubiquitous contaminants of concern in tap water. Chlorine is the most frequently used disinfectant worldwide, and trihalomethanes (THMs) are usually the most prevalent by-products of chlorination. The four common THMs, depending either on the chlorine or the bromine incorporation, are chloroform (TCM), bromodichloromethane (BDCM), dibromochloromethane (DBCM), and bromoform (TBM). THM4 stands for the sum concentration of the 4 individual species of TMHs. Long-term exposure to disinfection by-products has been associated with an increased risk of bladder cancer [1]. 

Bromate is a DBP formed by ozonation of water containing bromide. Bromate induces neurotoxicity in adults, and some evidences show a possible effect on thyroid hormone levels [2]. Bromate has also been found to be carcinogenic in male rats [3]. MX (3-chloro-4-(dichloromethyl)-5-hydroxy-2(5H)-furanone) is a DBP of a great concern given a strong mutagenic activity and is present in drinking water supplies [4] and multisite carcinogenicity in rats [5]. Hebert and colleagues [6] classified 110 nonregulated emerging DBP on the basis of their potential impact on public health; MX obtained the 5th position due to its high toxicity, regardless of the low concentrations usually found in water. According to the U.S. Environmental Protection Agency [7], MX's cancer potency was more than 6000-fold higher than that of chloroform and about 100-fold higher than that of BDCM.

Filtering and boiling are common activities in the household that modify DBP exposure through ingestion. There are a number of studies evaluating different heating, boiling, and filtering devices [8–15]. However, the use of a microwave has never been evaluated. Although the effect of filtering on MX levels has also been examined [12], the effect of boiling remains unknown. The change of bromate levels when filtering or boiling remains to be examined. Microwaves are electrical appliances of common use nowadays, and their effect on DBP levels is still unknown.

We have conducted this study in the city of Barcelona; its water is very unusual as the water from Llobregat River, the main supplier of the city, is high in bromide ion, and therefore high concentrations of brominated DBP are expected in the finished water. Generally, waters higher in chlorinated rather than brominated compounds are more common worldwide. Also, the water availability in the Mediterranean area varies within the season, with typically dry summers. This leads to a higher concentration of organic matter in the water, and along with higher temperatures, the doses of disinfectants to treat the water increase. All these accentuate the problem with DBP formation. The aim of this study is to evaluate the effect of boiling (microwave, saucepan, and kettle) and filtering (reverse osmosis and organic carbon jar-type filter) on trihalomethanes, MX, and bromate levels in a water with a high content of brominated DBP. 

## 2. Methods

### 2.1. Study Area

The experiments were conducted using water samples from Barcelona tap water in May and June 2009. Samples were taken at CREAL's tap water, in La Barceloneta neighbourhood, where tap water is a mixture of water from three different treatment plants from two different surface sources (Llobregat River and Ter River). The precise percentage of water coming from each source is unknown. However, the Llobregat River, which supplies a very high percentage of water in the study area, is rich in organic matter and bromine due to the discharges of salt mines upstream, in Cardener and Llobregat rivers themselves. Treatment processes for this water include disinfection using chlorine gas and chlorine dioxide, ozonation, and treatment with granulated activated carbon (GAC) filtration. The water from Ter River undergoes through the same treatment except for ozonation. 

### 2.2. Filtering Design

A pitcher filter containing granulated activated carbon and ion exchange resin filter (Brita) and a household reverse osmosis filtering system (Columbia) were used. For the pitcher experiment, we used a new filter and two artificially worn out filters by previously filtrating 75 L and 150 L of water, respectively, (150 L is the maximum volume of use established by the manufacturer) so we could study the effect of the age of the filter in terms of usage. Before performing the experiments and to mimic reality and to allow resins to reactivate, 1.5 L of water was filtered every 15 minutes when aging the filters until 75 L and 150 L, respectively, were filtrated through the pitcher. The vials for analysis were filled up after performing the experiment. 

### 2.3. Boiling Design

The boiling experiment was performed using a 3000 W lidded electric kettle (capacity of 1.7 L), a 14 cm diameter saucepan with a Bunsen burner and a 800 W microwave oven. Once the water reached the boiling point, the source of heat was immediately turned off. The kettle switched automatically off when water reached the boiling point. The volume of water depended on the capacity of the device, and we performed the experiment twice for each device (for THM and for MX/Bromate) except for the kettle, where there was enough volume of treated water to fill up the vials and the bottles for analysis. For the microwave experiment, the boiling point was assumed when bubbles appeared visible through the door. The vials/bottles for analysis were filled up 5 minutes after taking the water away from the heating source. This was done in order to mimic the ingestion of hot drinks. 

### 2.4. Measurements

Baseline samples were collected after leaving tap water running for 5 minutes in order to stabilise the pH, chlorine, and conductivity. The baseline vials/bottles were left opened until the end of each experiment. Samples to measure trihalomethanes were collected in 40 mL amber glass vials sealed with TFE-faced silicone septum screw caps, without any headspace to avoid loss of volatile THM. Ammonium chloride buffer solution (0.7 g/40 mL sample) was added to quench further chlorine reactions. For MX and bromate measurements, samples were collected in 500 mL and 100 mL PE bottles, respectively. MX was acidified with HCl 1 M to pH 2, and oxidants were quenched with ammonium sulphate (50 mg/100 mL sample); oxidants in bromate samples were quenched with ethylenediamine (1 ml of 0.5% EDA/100 mL sample). Trihalomethanes were measured in quadruplicate samples, and MX and bromate were measured in duplicate samples. 

During all stages of the experiments, pH, conductivity, and free and total chlorine were measured. pH was measured with a portable waterproof pH tester. Conductivity was measured with a platinum cell portable device. Free and total chlorine were determined with a colorimetric disc checker method. Samples to measure trihalomethanes were stored and sent to the laboratory at +4°C and analysed within the following 48 h. Samples to measure MX and bromate were frozen at −20°C and sent to the laboratory in dry ice. 

### 2.5. Analytical Procedure

Trihalomethanes samples underwent liquid/liquid extraction (LLE) by using 40% anhydrous sodium sulphate enriched samples (w/v) and n-pentane. They were analysed by gas chromatography/electron capture detection (GC/ECD), and 1,2-dibromopropane was used as an internal standard. Measurements were performed at the Gipuzkoa Laboratory of Public Health in Euskadi, Spain. The Finnish National Public Health Institute in Finland conducted the analysis for bromate and MX. Methods used were slightly modified from those published earlier [16, 17]. In brief, bromate was analysed with ion chromatography using suppressed conductivity detection, and MX was analysed with gas chromatography high resolution mass spectrometry (for more details see appendices). 

## 3. Results

The volume of water needed for each experiment depended on the capacity of each device (Table 1). The number of minutes for the water to start boiling also varied for each experiment. Filtering decreased pH in both filtration experiments and the reduction was most pronounced after a new pitcher filter. However, all boiling experiments resulted in an increase of pH except for the microwave experiment when analysing THM, where pH remained constant. Conductivity also increased for all boiling experiments and decreased for the filtration experiments; however, there was a very high reduction for the reverse osmosis test, over 97%. Free and total chlorine were also reduced after all treatments. Water took different amounts of time to boil depending on the volume of water and the experiment performed.

The tap water used for all the experiments contained a high percentage of bromoform (62% of THM4), while chloroform represented only a 4%; the three bromine-containing THM4 accounted for 96% of the 4 THM studied (*n* = 20, not shown in tables). The mean concentrations for MX and bromate in tap water were 0.73 ng/L and 3.3 *μ*g/L, correspondingly (*n* = 10, not shown in tables). The change in DBP concentration after each of the boiling and filtering experiments is shown in Table 2 and Table 3, respectively. 

All experiments led to a decrease of the 4 individual THM concentrations. Among the three boiling devices, the highest THM4 reduction was observed for the microwave oven (97%) with a very high decrease of the three brominated forms. The kettle experienced the lowest reduction in concentration for THM4 (48%), and, along with the saucepan test, chloroform experienced a higher percentage of removal than the brominated forms. Reverse osmosis led to the highest THM4 reduction among the evaluated filtering experiments (97%), with almost a 99% reduction for the bromine-containing species. However, chloroform concentration was only reduced by 56%. The pitcher filter had high percentages of THM4 reduction (89%) when the filter was new compared to both aged filters (76% for 75 L aged filter, and 74% for 150 L aged filter). Chloroform had higher removals than bromine-containing THM4 for the new and the 75 L aged filter but not for the older filter (150 L). 

Bromate showed a less consistent pattern, and concentration increased after boiling water in a saucepan and filtering through a pitcher filter with 75 L of previous usage. MX concentration decreased below the limit of quantification (LOQ) after all experiments.

## 4. Discussion

The water used for the experiments contained high concentrations of bromoform and brominated THM4. Water coming from Llobregat River, one of the two main suppliers of water in the city of Barcelona, is high in bromide due to the discharges of salt mines in Cardener and Llobregat rivers. Ventura and Rivera [18] showed that levels of bromide in water before and after Cardona mines increased substantially (0.01 and 1.98 mg/L Br, resp.). Bromide is not removed from water during a conventional treatment, so high levels of brominated-DBP were expected. In general, a high reduction of THM4 and MX was observed for all experiments. The highest elimination of brominated trihalomethanes was observed during filtering and chloroform during boiling the water, except for the microwave oven experiment and the newer filters of the pitcher filter. The concentration of bromate, a nonvolatile compound, was highly decreased with reverse osmosis and the kettle experiment, but the other devices did not show a significant effect on its concentration. However, the increases in the concentration of bromate (21% at maximum) are within the range of uncertainty of the measurement at the studied concentrations.

This is, to our knowledge, the first study evaluating the effect of heating water in a microwave oven on DBP levels, and this device is commonly used nowadays. The high reduction of THM4 for this test, especially for the brominated forms, could be due to the agitation of the water [11], as big bubbles were created when water started boiling. Moreover, in microwave heating, temperature can rise much faster than in conventional heating, and energy is not homogeneously dissipated; therefore, “hot spots” can occur [19], and some zones of the water mass can reach higher temperatures than others. Water took a long time to boil in the microwave oven (7 minutes for THM and 17 min for MX/bromate) due mainly to the large volume of water. Also, it was difficult to determine the precise moment of boiling, as we determined it by watching through the door. These, along with the fact that microwave ovens are sealed environments and the vapour pressure for all the compounds should be assessed, the results obtained may be difficult to interpret. 

Lower reductions for the kettle experiment could be due to the presence of a lid as it was a semisealed environment. Regarding this, similar THM4 reductions were found in other studies. A 64%, 93%, and 98% reduction of THM4 was observed after boiling chlorinated water in a kettle for 1, 2, and 5 minutes, respectively [13]. Batterman and colleagues [9] found that volatilization losses approached 67% when water was boiled in a kettle even for short periods of time (81% for TCM, 73% for BDCM, 62% for DBCM, and 58% for TBM). However, the volume of water used for this test was 1.05 L, which could allow a higher agitation of the water inside the kettle. This could be linked to the fact that higher reductions can occur when higher percentages of chlorinated compounds, which have a lower boiling point than brominated ones [10, 13], are present in water. Chloroform's boiling point is 60.3°C, and the boiling point rises as the degree of bromination rises (90°C for BDCM, 119°C for DBCM, and 150°C for bromoform). However, other authors reported higher removals [14] as high as 98% of the THM4 by heating water in a kettle up to the boiling point.

When water boiled in the saucepan, and taking into account that the heat source was not very powerful and that it took a long time (10 min for THM and 25 for MX/bromate), the bubbles formed were very small so there was little agitation. Generally, when boiling water in an open environment as in the test, the reduction of THM4 occurs mainly due to volatilization; therefore, these compounds can potentially be inhaled. 

The concentration of MX decreased during boiling, although previous studies showed no reduction. A Russian study [12] found that MX concentration remained unchanged when boiling very high chloroform-containing water (THM4 = 204 *μ*g/L, 97% chloroform). When waters are high in free chlorine, MX, which is a nonvolatile compound, might form and disappear at the same rate. However, in the water used for our experiments, chloroform only represented a very small percentage of total THMs, while bromoform especially and also BDCM and DBCM were clearly higher in concentration. 

Reverse osmosis appeared to be a very good method to eliminate brominated DBP, which agrees with previous studies [14]. The efficiency of granulated activated carbon increases with the number of bromine atoms in the molecule [18] and also because of their low solubility in water [14] which makes them more amenable to carbon adsorption. Chloroform had such high removals when using a pitcher filter (91% for the new filter), in comparison to the reverse osmosis system (56% reduction), mainly due to the agitation of the water when the pitcher was filled and also due to the water dropping from the filter to the pitcher itself. These results for the new filter are consistent with other studies despite reporting higher chloroform removals for older filters [12, 14, 20]. However, other studies showed lower reductions for THM4 [8]. In the present study, filtering water through previously aged filters reduced efficiency about 15% for the bromine-containing species due to the presence of previously filtrated particles which blocked the micropores in the activated carbon and the usage of the resins. MX was previously reported to greatly decrease after a pitcher filtration [12], and this is consistent with our results. Conductivity and pH decreased in the filtering experiments, especially for the pitcher filter, due to the removal of molecules and ions that could act as buffer solution. The change of pH in both filtration tests could involve a modification of the DBP mixture proportions. In fact, a decrease in pH reduces the formation of THM, but other DBP can actually form [21].

The low baseline levels of MX limit the evaluation of the actual concentration reduction, as the concentration of MX after all treatments was below the LOQ. To calculate a percentage of change, we would have had to impute for example, half of the LOQ and assume that value as the concentration of MX after the treatment. However, levels could have been either higher or lower than half of the LOQ, and this would affect the real percentage of change. We also have to consider that in waters high in bromide, it is likely that brominated analogues of MX (BMXs) are formed together with brominated THMs [22]. However, due to the lack of certified commercial model compounds for BMXs and the substantial difficulties experienced with the instability of BMXs in the GC liner and column, which makes their accurate quantification very challenging [17], we are not considering them here. 

Samples were taken in quadruplicates for THM4 and in duplicates for MX and bromate, and results show the mean. Ideally, these experiments should have been performed several times, and the percentage of change would be the mean of all experiments. Also, the number of samples should be higher before and after each experiment in order to have a more accurate percentage of change. There is also the need to mention that the amount of water used for each experiment can affect the percentage of DBP change, mainly in the boiling experiments. Smaller amounts of water can potentially experience higher reductions as the agitation of the water will be higher. Also, we did not consider the volume of water loss during the experiments, which could also affect the concentration of the DBP analysed. These experiments were only performed for THM, MX, and bromate but we do not know the change that would occur for other compounds not analysed in this study or for combinations of them as DBPs are a complex mixture. Regarding MX and its brominated analogues, one option in further studies might be to measure the change in Ames mutagenicity assay as a surrogate measure of change in BMX concentrations which have mutagenic activity comparable to that of MX [23].

These experiments will allow us to develop in the future individual exposure correction factors that will be based on the different household water uses. These factors, together with the patterns of use and consumption of water for a specific population (ingestion, hygienic and leisure habits, etc.) and the environmental levels of the different DBPs will generate exposure indices. These indices will be applied to epidemiological studies to evaluate some potential adverse health effects.

## 5. Conclusions 

Treating water at the household can greatly impact the concentration of disinfection by-products. Filtering and boiling tap water reduces the concentrations of trihalomethanes and MX. Among trihalomethanes, the largest reduction in chloroform is produced by boiling in a microwave or a saucepan, while brominated THMs are mostly reduced by reverse osmosis and a pitcher-type filter. The concentrations of bromate also decrease by reverse osmosis and the pitcher filter. However, further studies with this highly brominated water considering different pH, volumes of the water, and time taken for the experiment are needed. This knowledge will help to minimise the exposure misclassification for individual-level DBP exposure measures in epidemiological studies. 

## Figures and Tables

**Table 1:** Characteristics of the water before and after each treatment (volume refers to treated water; time refers to minutes to complete the experiment). These values represent only one measurement each. The characteristics of the water for the three different groups of DBP are presented together when the volume of water was sufficient to take samples for all the studied DBP at once.

Treatment	Measurements	Stage	pH	Conduct.	Free Cl	Total Cl	Volume	Time
				(*μ*S/cm)	(mg/L)	(mg/L)	(L)	(min)
Boiling								
Kettle	THM/MX/bromate	Before	7.89	1718	0.44	0.56	1.7	4
		After	7.99	1800	0.02	0.08		
Microwave	THM	Before	8.00	2100	0.10	0.34	0.4	7
		After	8.00	2400	0.02	0.04		
	MX/bromate	Before	8.02	1719	0.10	0.30	1.2	17
		After	8.50	1881	0.04	0.06		
Saucepan	THM	Before	7.96	2070	0.40	0.50	0.4	10
		After	8.12	2400	0.02	0.14		
	MX/bromate	Before	8.04	1713	0.50	0.50	1.4	25
		After	8.36	1823	0.00	0.10		

Filtering								
Reverse osmosis^a^	THM	Before	7.91	1722	0.20	0.46	—	—
		After	6.68	58.6	0.04	0.04		
	MX/bromate	Before	7.85	1707	0.26	0.44	—	—
		After	6.93	60	0.04	0.06		
Activated carbon pitcher-type filter	THM/MX/bromate	Before	8.21	1730	0.10	0.24		
		After 1 L	5.39	1438	0.08	0.10	1.5	
		After 75 L	6.38	1424	0.08	0.12	1.5	—
		After 150 L	6.80	1571	0.08	0.22	1.5	

^a^The variable volumes of water and amount of time are not applicable for this experiment.

**Table 2:** Mean concentration, standard deviation (only for THM4), minimum and maximum of the 4 studied THM^a^ (*n* = 4), and mean and value of the two samples for MX^b^ and bromate^c^ (*n* = 2) before and after the boiling experiments. Percentage of change in bold.

	Kettle		Microwave		Saucepan	
	Before	After	Before	After	Before	After
Chloroform (TCM)						
Mean (SD)	7.30 (0.24)	2.25 (0.11)	2.13 (0.07)	0.33 (0.32)	2.79 (0.07)	0.61 (0.45)
Min–max	(6.97–7.50)	(2.13–2.36)	(2.04–2.21)	(0.09–0.78)	(2.72–2.88)	(0.37–1.29)
% change		** −69 **		** −84 **		** −78 **
Bromodichloromethane (BDCM)						
Mean (SD)	15.37 (0.33)	4.97 (0.23)	5.61 (0.18)	0.16 (0.03)	7.88 (0.48)	1.82 (0.13)
Min–max	(14.90–15.59)	(4.71–5.22)	(5.42–5.84)	(0.14–0.20)	(7.18–8.22)	(1.64–1.95)
% change		** −68 **		** −97 **		** −77 **
Dibromochloromethane (DBCM)						
Mean (SD)	36.54 (0.78)	17.84 (0.72)	22.06 (0.51)	0.53 (0.10)	31.27 (0.89)	9.18 (0.69)
Min–max	(35.43–37.26)	(17.01–18.55)	(21.42–22.68)	(0.45–0.67)	(30.01–32.04)	(8.26–9.75)
% change		** −51 **		** −98 **		** −71 **
Bromoform (TBM)						
Mean (SD)	66.62 (1.04)	39.98 (1.43)	78.96 (1.78)	2.02 (0.42)	93.37 (0.72)	33.78 (2.03)
Min–max	(65.36–67.90)	(38.49–41.53)	(76.70–81.04)	(1.72–2.64)	(92.65–94.35)	(31.03–35.57)
% change		** −40 **		** −97 **		** −64 **
Sum of 4 THMs (THM4)						
Mean (SD)	125.85 (2.32)	65.05 (2.49)	108.76 (2.53)	3.04 (0.85)	135.32 (1.87)	45.4 (2.45)
Min–max	(122.66–128.19)	(62.34–67.66)	(105.58–111.77)	(2.40–4.29)	(133.05–137.39)	(42.22–47.60)
% change		** −48 **		** −97 **		** −66 **
MX						
Mean	0.9	<0.5	0.60	<0.5	0.90	<0.5
Min–max	(0.7–1.0)	(all < 0.5)	(0.60–0.60)	(all < 0.5)	(0.7–1.1)	(all < 0.5)
% change*		—		—		—
Bromate						
Mean	3.9	2.3	3.9	3.6	3.7	4.4
Min–max	(3.6–4.1)	(2.3)	(3.6–4.1)	(3.2–3.9)	(3.2–4.1)	(4.1–4.7)
% change		** −40 **		** −8 **		** 21 **

^a^THM4 and all 4 species: concentration in *μ*g/L, limit of quantification of 0.4 *μ*g/L, all means *n* = 4.

^b^MX: concentration in ng/L, limit of quantification of 0.5 ng/L, all means *n* = 2.

^c^Bromate: concentration in *μ*g/L, limit of quantification of 0.5 *μ*g/L, all means *n* = 2.

*Percentages of change when values were under the limit of quantification (LOQ) for MX are not reported due to high uncertainty.

**Table 3:** Mean concentration, standard deviation (only for THM4), minimum and maximum of the 4 studied THM^a^ (*n* = 4), and mean and value of the two samples for MX^b^ and bromate^c^ (*n* = 2) before and after the filtration experiments. Percentage of change in bold.

	Reverse osmosis		Activated carbon pitcher-type filter			
				1 L	75 L	150 L
	Before	After	Before	After	After	After
Chloroform (TCM)						
Mean (SD)	7.3 (0.06)	3.21 (0.04)	3.46 (0.14)	0.31 (0.03)	0.70 (0.03)	1.17 (0.48)
Min–max	(7.25–7.39)	(3.17–3.27)	(3.31–3.61)	(0.27–0.33)	(067–0.74)	(0.88–1.89)
% change		** −56 **		** −91 **	** −80 **	** −66 **
Bromodichloromethane (BDCM)						
Mean (SD)	15.09 (0.18)	0.58 (0.04)	8.75 (0.31)	0.99 (0.10)	1.99 (0.05)	2.20 (0.07)
Min–max	(14.94–15.33)	(0.56–0.64)	(4.48–9.13)	(0.88–1.09)	(1.92–2.04)	(2.12–2.28)
% change		** −96 **		** −89 **	** −77 **	** −75 **
Dibromochloromethane (DBCM)						
Mean (SD)	35.48 (0.29)	0.10 (0.12)	27.25 (0.91)	2.82 (0.24)	6.15 (0.17)	6.67 (0.15)
Min–max	(35.08–35.72)	(0.02–0.27)	(26.40–28.26)	(2.58–3.11)	(5.90–6.26)	(6.47–6.80)
% change		** −99 **		** −90 **	** −77 **	** −75 **
Bromoform (TBM)						
Mean (SD)	69.44 (0.89)	0.18 (0.25)	64.76 (1.86)	6.91 (0.65)	16.42 (0.37)	16.69 (0.19)
Min–max	(68.71–70.58)	(0.03–0.55)	(62.68–66.64)	(6.37–7.78)	(16.15–16.97)	(16.53–16.97)
% change		** −99 **		** −89 **	** −75 **	** −74 **
Sum of 4 THMs (THM4)						
Mean (SD)	127.31 (0.87)	4.07 (0.41)	104.23 (3.18)	11.04 (1.00)	25.26 (0.56)	26.74 (0.72)
Min–max	(126.64–128.59)	(3.82–4.68)	(101.07–107.64)	(10.20–12.31)	(24.64–26.01)	(26.00–27.65)
% change		** −97 **		** −89 **	** −76 **	** −74**
MX						
Mean	0.8	<0.5	0.6	<0.5	<0.5	<0.5
Min–max	(0.7–0.8)	(all < 0.5)	(0.5–0.6)	(all < 0.5)	(all < 0.5)	(all < 0.5)
% change*		—		—	—	—
Bromate						
Mean	2.3	0.5	2.90	2.6	3.10	2.55
Min–max	(2.2–2.4)	(all <0.5)*	(2.9-2.9)	(2.3–2.8)	(2.8–3.4)	(2.5–2.6)
% change		—		** −12 **	** 7 **	** −12 **

^a^THM4 and all 4 species: concentration in *μ*g/L, limit of quantification of 0.4 *μ*g/L, all means *n* = 4.

^b^MX: concentration in ng/L, limit of quantification of 0.5 ng/L, all means *n* = 2.

^c^Bromate: concentration in *μ*g/L, limit of quantification of 0.5 *μ*g/L, all means *n* = 2.

*Percentages of change when values were under the limit of quantification (LOQ) for MX and bromate are not reported due to high uncertainty in the estimate.

## Appendices

### A. MX Analysis

Ammonium sulphate (50 mg/100 mL of sample) was added to the samples to quench the oxidants, and pH of water samples was adjusted to 2 to stabilize the MX to a stable ring form. Samples (100 mL) were pumped with tubing pumps through Waters Sep-Pak Plus tC_18_ and HLB-Plus solid phase extraction cartridges. Sep-Pak Plus tC_18_ retains impurities like humic compounds, and HLB Plus retains MX. MX was eluted from HLB Plus cartridges with 4 mL of acetone that was evaporated to dryness.

Internal standard 13C-245-trichlorophenol (125 *μ*L of 4.0 ng/mL solution in isopropanol), 125 *μ*L of 4% H_2_SO_4_-isopropanol, 100 *μ*L of nonane, and 200 *μ*L of hexane were added to samples. Samples were placed for 1 hour to 85°C to isopropylate the analytes. After cooling, 2 mL of ultrapure water and 4 mL of hexane were added, and samples were mixed. Hexane phase was separated, washed with 1 mL of ultrapure water, dried with sodium sulphate, evaporated to a volume of about 500 *μ*L, and transferred to autosampler vials. One ng of recovery standard PCB 30 was added to autosampler vials, and solvent was concentrated to final volume of about 50 *μ*L.

Isopropyl derivative of MX was analysed with gas chromatograph (Hewlett Packard 6890) coupled to high resolution mass spectrometer (Waters Autospec Ultima). Column used was DB-5MS (Agilent Technologies: 30 m, i.d. 0.25 mm, 0.25 *μ*m).

Final results were calculated by addition of a known amount of MX to a second 100 mL sample aliquot and by means of this addition calculating the original concentration in the sample. Limit of quantification for MX was 0.5 ng/l. Uncertainty of measurement at 10 ng/l is ±50% [17].

### B. Bromate Analysis

To prevent further bromate formation, ethylenediamine was pipetted to the bottom of sample bottles (1ml of 0.5% EDA/100 mL sample), which quenches residual oxidants in the samples.

Cation exchange cartridges were used for online removal of chloride that elutes right after bromate by pumping samples from autosampler through silver and proton cartridges to 400 *μ*L sample loop. Silver cartridge precipitates chloride and other halide ions, and proton cartridge removes silver ions leached from the silver cartridge. Ion chromatograph used was Dionex DX-600, columns were Dionex AG11-HC and AS11-HC, and suppressor was Dionex ASRS-ULTRA operated in the recycle mode. Bromate was eluted through columns with KOH eluent. Details of ion chromatographic conditions can be found elsewhere [16].

Internal standard, trifluoroacetic acid, was used in the quantification of bromate. Method is accredited. Limit of quantification for bromate is 0.5 *μ*g/l. Uncertainty of measurement at 10 *μ*g/l is ±20%.

## Abbreviations

DBP: Disinfection by-products
THM: Trihalomethanes
MX: 3-Chloro-4-(dichloromethyl)-5-hydroxy-2(5H)-furanone.

## References

[1] Villanueva CM, Cantor KP, Cordier S (2004). Disinfection byproducts and bladder cancer: a pooled analysis. *Epidemiology*.

[2] Crofton KM (2006). Bromate: concern for developmental neurotoxicity?. *Toxicology*.

[3] U.S. Environmental Protection Agency Public health goals for chemicals in drinking water: bromate.

[4] Hemming J, Holmbom B, Reunanen M, Kronberg L (1986). Determination of the strong mutagen 3-chloro-4-(dichloromethyl)-5-hydroxy-2(5H)-furanone in chlorinated drinking and humic waters. *Chemosphere*.

[5] Komulainen H, Kosma VM, Vaittinen SL (1997). Carcinogenicity of the drinking water mutagen 3-chloro-4- (dichloromethyl)-5-hydroxy-2(5H)-furanone in the rat. *Journal of the National Cancer Institute*.

[6] Hebert A, Forestier D, Lenes D (2010). Innovative method for prioritizing emerging disinfection by-products (DBPs) in drinking water on the basis of their potential impact on public health. *Water Research*.

[7] U.S. Environmental Protection Agency Office of Environmental Health Hazard Assessment Reproductive (OEHHA). Cancer Hazard Assessment Section.

[8] Gibbons J, Laha S (1999). Water purification systems: a comparative analysis based on the occurrence of disinfection by-products. *Environmental Pollution*.

[9] Batterman S, Huang AT, Wang S, Zhang L (2000). Reduction of ingestion exposure to trihalomethanes due to volatilization. *Environmental Science and Technology*.

[10] Wu WW, Benjamin MM, Korshin GV (2001). Effects of thermal treatment on halogenated disinfection by-products in drinking water. *Water Research*.

[11] Li XZ, Sun JM (2001). Further formation of trihalomethanes in drinking water during heating. *International Journal of Environmental Health Research*.

[12] Egorov AI, Tereschenko AA, Altshu LM (2003). Exposures to drinking water chlorination by-products in a Russian city. *International Journal of Hygiene and Environmental Health*.

[13] Krasner SW, Wright JM (2005). The effect of boiling water on disinfection by-product exposure. *Water Research*.

[14] Weinberg HS, Pereira VRPJ, Singer PC, Savitz DA (2006). Considerations for improving the accuracy of exposure to disinfection by-products by ingestion in epidemiologic studies. *Science of the Total Environment*.

[15] Rahman MD, Driscoll T, Clements M, Armstrong BK, Cowie CT (2011). Effects of tap water processing on the concentration of disinfection by-products. *Journal of Water and Health*.

[16] Rantakokko P, Mustonen S, Vartiainen T (2003). Suppressor current switching: a simple and effective means to reduce background noise in ion chromatography. *Journal of Chromatography A*.

[17] Rantakokko P, Yritys M, Vartiainen T (2004). Matrix effects in the gas chromatographic-mass spectrometric determination of brominated analogues of 3-chloro-4-(dichloromethyl)-5-hydroxy-2(5H)-furanone. *Journal of Chromatography A*.

[18] Ventura F, Rivera J (1985). Factors influencing the high content of brominated trihalomethanes in Barcelona’s water supply (Spain). *Bulletin of Environmental Contamination and Toxicology*.

[19] Berlan J (1995). Microwaves in chemistry: another way of heating reaction mixtures. *Radiation Physics and Chemistry*.

[20] Eslinger SA, Weinberg HS Estimating average daily exposure to disinfection by-products in drinking water by examining alternate ingestion pathways.

[21] Hansen KMS, Willach S, Antoniou MG, Mosbæk H, Albrechtsen HJ, Andersen HR (2012). Effect of pH on the formation of disinfection byproducts in swimming pool water—is less THM better?. *Water Research*.

[22] Suzuki N, Nakanishi J (1995). Brominated analogues of MX (3-chloro-4-(dichloromethyl)-5-hydroxy-2(5h)-furanone) in chlorinated drinking water. *Chemosphere*.

[23] LaLonde RT, Bu L, Henwood A, Fiumano J, Zhang L (1997). Bromine-, chlorine-, and mixed halogen-substituted 4-methyl-2(5H)- furanones: synthesis and mutagenic effects of halogen and hydroxyl group replacements. *Chemical Research in Toxicology*.

